# P(LMA-co-tBMA-co-MAA) Copolymers Bearing Amphiphilic and Polyelectrolyte Characteristics: Synthetic Aspects and Properties in Aqueous Solutions

**DOI:** 10.3390/polym17111473

**Published:** 2025-05-26

**Authors:** Anastasia Balafouti, Stergios Pispas

**Affiliations:** 1 Theoretical and Physical Chemistry Institute, National Hellenic Research Foundation, 48 Vassileos Constantinou Ave, 11635 Athens, Greece; ampalaf@eie.gr; 2 Department of Chemistry, National and Kapodistrian University of Athens (NKUA), 15784 Athens, Greece

**Keywords:** amphiphilic random copolymers, polyelectrolytes, RAFT, hydrolysis, nanoaggregates, post-polymerization modification, self-assembly, pH responsive

## Abstract

In this study, we explore the design of novel random poly(lauryl methacrylate-co-tert-butyl methacrylate-co-methacrylic acid), P(LMA-co-tBMA-co-MAA) copolymers via the RAFT copolymerization of LMA and tBMA followed by the selective hydrolysis of tBMA segments. For the molecular characterization of the novel copolymer, a series of physicochemical techniques were implemented, including size exclusion chromatography (SEC), proton nuclear magnetic resonance (^1^H-NMR) and attenuated total reflectance–Fourier transform infrared (ATR–FTIR) spectroscopy. Our experimental results confirmed the successful synthesis of the targeted copolymers. The compositions were in accordance with the targeted differing fraction of hydrophobic tBMA/LMA elements, and hydrolysis resulted in at least 64% conversion to hydrophilic MAA units. The copolymers, bearing both an amphiphilic character and polyelectrolyte properties while being composed of randomly distributed monomeric segments of biocompatible materials, were subsequently investigated in terms of their self-assembly behavior in aqueous solutions. Dynamic light scattering and fluorescence spectroscopy experiments demonstrated the formation of self-assembled nanoaggregates (average hydrodynamic radii, R_h_ < 100 nm) that formed spontaneously, having low critical aggregation concentration (CAC) values (below 3.5 × 10^−6^ g/mL), and highlighted the feasibility of using these copolymer systems as nanocarriers for biomedical applications.

## 1. Introduction

Since key scientific and technological advancements in polymer chemistry allow for the production of biocompatible polymers with predetermined topologies, compositions and behaviors in solutions, polymer nanoassemblies have recently emerged as intriguing multiadaptive alternatives in order to optimize the biodistribution of therapeutic agents [1,2]. Amphiphilic copolymers serve as protagonists in this field. The covalent bonding between versatile monomer units combined with the contrasting solubility properties within macromolecular polymer chains, or with the surrounding environment, results in the spontaneous formation of patterned morphologies through specific non-covalent interactions that tend to minimize free energy. Hydrophobic effects, electrostatic interactions or van der Waals forces are some of the predominant interactions that guide this self-assembly process, which is manipulated by a number of factors including the primary structure of the polymer (chain length, composition, monomer sequence distribution and macromolecular architecture/topology), the solubilization method applied and the inherent physicochemical parameters of the solution [3,4]. While enhancing bioavailability, site-specific targeting and pharmacokinetic properties, such macromolecular morphologies at the nanoscale have shown great potential for the delivery of drugs [2], genes [5], diagnostic agents [6] and vaccines [7].

Poly(methacrylic acid), PMAA, is a biocompatible weak anionic polyelectrolyte with bioadhesive properties, commonly explored for oral drug delivery systems [8,9]. Alongside commercial formulations of MAA and various kinds of methacrylate (MA) copolymers, P(MAA-co-MA) are utilized as oral pharmaceutical tablet coatings that improve controlled drug release [10,11]. In aqueous solutions, the conformation of PMAAs and PMAA copolymers depends on the fraction of dissociated ionic groups; hence, the polymers present pH sensitivity. At physiological pHs, as well as at high pH values over their pK_a_, ionizable carboxylic acid groups are deprotonated. Strong electrostatic repulsions occur between them, causing the polymer chains to expand and expose more polymer molecules to interact with water molecules, leading to greater hydrophilicity. Conversely, at low pH values, where the carboxylic acid groups remain protonated, polymer chains become more hydrophobic [12]. pH responsiveness leverages the physiological pH gradients present within the different microenvironments of the human body to achieve targeted functionality [13].

This property is investigated today in relation to the development of MAA copolymer nanoparticles (NPs). Disassembly or dissolution at target pH values is adjustable by tailoring the combination of polymer components, suggesting high application versatility. For instance, Luo and coworkers developed copolymers of MAA and ethylene glycol (EG) based on three covalently linked blocks PMAA-b-PEG-b-PMAA as a stable molecular platform for drug delivery to lesion sites. Self-assembly studies in aqueous media revealed the presence of core–shell micellar structures where pH sensitive PMAA constitutes the core, surrounded by a highly hydrophilic PEG shell. Increasing copolymers’ molecular weight was found to decrease their micellar size (range 18 to 89 nm) and critical micelle concentration. Micelles, which are able to encapsulate hydrophobic drug prednisone in their core, displayed pH-dependent phase transition at a pH value of about 5.2, with stable micellization behavior in the 4.8 to 7.4 pH range, allowing for controlled drug release by diffusion due to the dissociation of PMAA chains [14]. A different work, by Kamenova and her team, developed analogous PMAA-b-PCL-b-PMAA block copolymers based on MAA instead of PEG, while hydrophobic ε-caprolactone (CL) was used as a copolymer component, aiming for the oral delivery of the anti-inflammatory drug resveratrol. The copolymers demonstrated the ability to self-assemble and incorporate the drug into spherical nanoscale structures with a mean hydrodynamic diameter of 78 nm which exhibited pH-sensitive behavior. The in vitro evaluation of the controlled drug release revealed that the drug was also located in the corona, while almost complete release was possible at both pH 6.8, simulating the intestinal environment, and pH 1.2, simulating the gastric environment [15].

Embracing a hydrophobic compartment in the polymer chain, apart from inducing the micellization process, may prompt an extra advantage upon drug delivery, that being higher efficiency in the disruption of lipid cell membranes through hydrophobic interactions. In the pivotal study by Thomas and colleagues, examining the connection between anionic charge and hydrophobicity utilizing poly (ethyl acrylic acid-co-MAA), P(EAA-co-MAA), copolymer, it was observed that, as the mol.% of MAA decreased from 51% to 0%, vesicle suspensions were clarified at a progressively higher pH range of 5.8–6.6, while PMAA did not seem to clear these suspensions even at a pH of 5.5. Determining the degree of polymer ionization via pH titration at the critical pHs proved that copolymers with higher hydrophobic contents promoted membrane dissolution, despite their higher degrees of macromolecular ionization [16].

Another crucial factor regarding application efficiency is monomer sequence distribution. In general, regardless of hydrophilic corona surface charge, research interest is centered around amphiphilic A-B- or A-B-A-type copolymers which feature systematically arranged distinct monomer species that form hydrophilic and hydrophobic homopolymer blocks linked in sequence [17]. Amphiphilic block copolymers self-assemble into structures with hydrophobic cores and hydrophilic coronas of a high definition. The nanosystems shaped through microphase separation usually comprise thermodynamically stable uniform micelles, the size and shape of which can be easily tuned by the relative volume fraction of the immiscible blocks [18]. However, in comparison to copolymers bearing a random monomer distribution, synthesis is not feasible by a one-pot polymerization reaction. Hence, in recent decades, drug carrier application studies have expanded to include amphiphilic copolymers of diverse topologies with rather interesting results. As an example, a systematic study of amphiphilic copolymers of hydrophilic poly(N-(2-hydroxypropyl)-methacrylamide modified with randomly distributed hydrophobic lauryl methacrylate, P(HPMA-co-LMA), revealed that their self-assembly in aqueous media led to the formation of aggregates that exhibited increased cellular uptake compared with P(HPMA) homopolymers and P(HPMA)-b-P(LMA) block copolymers [19].

Recently, Wannasarit et al. reported the fabrication of acetylated dextran-grafted P(MAA-co-LMA) copolymers in order to resemble a nonenveloped oncolytic virus for antitumor therapeutics. The study included a systematic investigation of the effects of alkyl chain length on membrane permeability. Employing a 140 to 10 MAA to hydrophophic methacrylate segment ratio in their copolymerization of MAA with methacrylates of 4 (butyl methacrylate, BMA), 12 (LMA) and 19 (stearyl methacrylate, SMA) carbons, followed by polymer–lipid membrane interaction characterization by a hemolysis assay, the researchers concluded LMA to be the optimal material for pH-dependent endosomolytic activity [20]. The random topological combination of MAA and LMA has also been employed to constitute the core of potent vaccine carriers prepared by the self-assembly of poly(ethylene glycol) methyl ether methacrylate-co-pyridyl disulfide methacrylate (PEGMA-co-PDSM)–block-(LMA-co-MAA) copolymers, where P(PEGMA-co-PDSM) serves as the hydrophilic corona for antigen conjugation and MAA-co-LMA serves as the protective core for the vaccine adjuvant Imiquimod (IMQ). On this occasion, 50 mole% LMA (among the 25–75% range) copolymers demonstrated favorable properties, presenting the highest IMQ loading [21].

It is obvious that various amphiphilic copolymers of MAA and LMA possess attractive functionalities in terms of the design of therapeutic nanocarrier systems, while every copolymer species presents a unique nanosystem. To our knowledge, a report on their assembly in aqueous media including a high percentage of LMA and a nonspecifically functioning conjugate is missing. Additionally, there is an obstacle concerning their preparation, arising from the high polar incompatibility between these monomers [19,20,21,22]. Herein, we report the synthesis of random poly(lauryl methacrylate-co-tert-butyl methacrylate-co-methacrylic acid), P(LMA-co-tBMA-co-MAA), copolymers of high-LMA consistency, utilizing the reversible addition fragmentation chain transfer (RAFT) copolymerization [23] of LMA and tBMA and the post-polymerization modification of tBMA segments by selective hydrolysis. This procedure puts emphasis on acquiring copolymers with narrow molecular weight distributions, which is a challenge when the free radical copolymerization of unsaturated carboxylic acids is employed, even when a reversible-deactivation radical polymerization (RDRP) technique like RAFT is applied. Moreover, P(MAA-co-tBMA) copolymers have already been proven to be nontoxic against the cells of the immune system [24]. We assumed that the fabrication of polyelectrolyte amphiphilic copolymers combining the three segments would facilitate the design of multifunctional nanosystems that consist of micellar structures where a prominently hydrophobic core is decorated with a negatively charged hydrophilic corona. Theoretically, these systems could comprise a tunable neat platform for hydrophobic, amphiphilic or cationic bioactive compound delivery. Considering all the above factors, we further proceeded to explore the self-assembly of amphiphilic polyelectrolyte copolymers in aqueous media.

## 2. Materials and Methods

### 2.1. Materials

All chemicals, including lauryl methacrylate (LMA), tert-butyl methacrylate (tBMA), monomethyl ether hydroquinone and butylated hydroxytoluene (inhibitors) removers, 2,2 azobisisobutyronitrile (AIBN), 4-cyano-4-(phenyl-carbonothioylthio)-pentanoic acid (CPAD), pyrene, 1,4-dioxane (99.8% pure), *n*-hexane, tetrahydrofuran (THF), trifluoroacetic acid (TFA), hydrochloric acid 36.5% (HCL), methanol, toluene, deuterated tetrahydrofuran (THF-d8), deuterated chloroform (CdCL_3_) and sodium hydroxide (NaOH), were supplied by Sigma Aldrich (St. Louis, MO, USA).

### 2.2. Synthesis of P(LMA-co-tBMA) Copolymers

Two P(LMA-co-tBMA) precursor copolymers (denoted as PC1 and PC2) were prepared via RAFT solution polymerization. The two copolymers differ only in targeted LMA to tBMA molar ratio, and each polymerization reaction was carried out for 24 h in a 25 mL round-bottom flask. The typical polymerization protocol for both cases is described below, with the details for (PC1/PC2) presented as such: LMA (0.6 g, 2.4 mmol/1 g, 3.9 mmol) and tBMA (1.4 g, 9.8 mmol/1 g, 7.0 mmol), formerly purified by inhibitor remover-filled columns, were mixed with the RAFT agent CPAD (55.8 mg, 0.2 mmol), recrystallized by the methanol initiator AIBN (16.4 mg, 0.1 mmol) and 1,4 dioxane (7.713 mL), creating a 20% *w*/*w* monomer solution. A magnetic stir bar was then added to the mixture and the flask was sealed with a rubber septum. The mixture was degassed under stirring by nitrogen purging for approximately 20 min. Purging was halted, and while confined under a nitrogen inert atmosphere with the aid of the septum, the solution was subsequently submerged in a thermostated and magnetically stirred oil bath, adjusted to 70 °C. After polymerization, the flask was removed from the oil bath and placed in a freezer at −20 °C to achieve rapid and homogenous polymerization termination. The frozen sealed solution was then left at room temperature, followed by exposure to air. A sample of the solution was characterized by SEC, which qualitatively confirmed the absence of unreacted monomers; hence, the polymer was separated by precipitation in an excess of a 10% *v*/*v* water to methanol solution. The precipitate was later collected after dilution in THF and vacuum drying for 48 h. Both precursor polymers were characterized by ^1^H-NMR and FTIR spectroscopy.

### 2.3. Selective Acid Hydrolysis of P(LMA-co-tBMA) Copolymers

Aiming towards the production of amphiphilic polyelectrolyte P(LMA-co-tBMA-co-MAA) copolymers, selective acidolysis of the tBMA groups was performed as described below, with the details for (PC1/PC2) given as such. (1.886 mL/1.078 mL) TFA (50-fold excess to tBMA) was added into a (20 mL/16 mL) THF dispersion of PC1 (1.0 g)/PC2 (0.8 g) under stirring. The mixture was left to react under stirring at room temperature in a sealed round-bottom flask that was completely covered from light. After seven days of hydrolysis reaction, the solutions were concentrated in a rotary evaporator to secure their purification from any traces of the byproduct gaseous isobutylene [25], and later collected after drying in a vacuum oven for 48 h.

### 2.4. Preparation of P(LMA-co-tBMA-co-MAA) Copolymer Nanoassemblies in Aqueous Solutions

The typical protocols for organic solvent displacement [26] and direct dissolution in aqueous media were employed to prepare stock aqueous solutions of the final produced amphiphilic copolymers (denoted as C1/C2, respectively). In all cases, the copolymer concentration was 5 × 10^−4^ g/mL, and distilled water of 0.1 M ionic strength (using appropriate amount of NaCl) was used as the aqueous medium. In the case of the solvent displacement protocol, a concentrated THF copolymer solution was rapidly injected into the aqueous medium under vigorous stirring and the generated mixture was stirred and heated while placed in a thermostated water bath until the complete evaporation of the organic solvent was achieved. Samples of the resulting solutions were subjected to pH manipulation by adding proper quantities of 0.1 M HCL and 1 M NaOH solutions.

### 2.5. Size Exclusion Chromatography (SEC)

In order to evaluate the molecular weight distributions of the synthesized copolymers, samples of the PC1/PC2 solutions after polymerization were diluted in THF at a concentration of approximately 1 mg/mL and passed through a Waters Corporation (Milford, MA, USA) SEC setup, equipped with a Waters 1515 isocratic pump, three μ-Styragel mixed-bed columns of 10^2^–10^6^ Å pore size and a Waters 2414 refractive index detector maintained at 40 °C. THF with 5% *v*/*v* triethylamine was used as the eluent at a flow rate of 1.0 mL/min at 30 °C. The setup was calibrated with narrow polystyrene standards and was performed with Breeze 2.0 Software (Waters Corporation, Milford, MA, USA).

### 2.6. Proton Nuclear Magnetic Resonance (^1^H-NMR) Spectroscopy

^1^H-NMR spectroscopy was utilized to assess the composition of the obtained copolymers. The experiments were performed on a Varian V300 MHz spectrometer operated by VNMRJ 2.2C Software (Palo Alto, CA, USA). The measured samples were solutions of the dry copolymers at an approximately 14 mg/mL concentration in CDCl_3_ or THF-d8. The chemical shifts in the recorded spectra were attributed in parts per million (ppm) relative to tetramethylsilane (TMS) as the internal standard, and overall spectra inspection was feasible using MestRe Nova 14.0.0 Software (Mestrelab Solutions, Bajo, Spain).

### 2.7. Fourier Transform Infrared (FTIR) Spectroscopy

The FTIR analysis of dry compressed solid copolymer samples was employed to further analyze the copolymers’ chemical structures. Measurements were carried out on a Bruker (Billerica, MA, USA) Equinox 55 spectrometer equipped with a single-bounce attenuated total reflectance (ATR) diamond accessory (Dura-Samp1IR II by SensIR Technologies, Danbury, CT, USA). Recorded spectra from 5000 to 500 cm^−1^ were obtained, taken from an average of 64 scans at 4 cm^−1^ resolution.

### 2.8. Fluorescence Spectroscopy

Fluorescence measurements were conducted on a Fluorolog-3 Jobin Yvon-Spex spectrofluorometer (model GL3-21, Horiba, Kyoto, Japan) to determine the critical aggregation concentration (CAC) of the C1/C2 amphiphilic copolymers in aqueous solutions. A typical fluorescence assay was carried out using pyrene as the sensitive probe [27], following the steps described below. At first, pyrene was dissolved in acetone to prepare a 1mM stock solution. Next, stock C1/C2 solutions in aqueous media were subjected to successive dilutions in order to create a new set of eleven copolymer solutions ranging in concentration from 5 × 10^−4^ to 5 × 10^−9^ g/mL. Then, an adequate quantity of the stock pyrene solution was added to each copolymer solution at a 1 µL/mL standard mixing ratio. The emerging solution series were left overnight in the absence of light so that the acetone solvent was eliminated by evaporation. The excitation wavelength was set to 335 nm and emission spectra were recorded in the 355–630 nm range.

### 2.9. Dynamic Light Scattering (DLS)

The self-assembly properties of the copolymers in aqueous media were examined by DLS measurements conducted on an ALV/CGS-3 photometer (ALV GmbH, Hessen, Germany). The set comprised a JDS Uniphase He-Ne 22 mW laser source (632.8 nm), a compact goniometer system with an Avalanche photodiode detector interfaced with an ALV/LSE-5003 light scattering electronics unit as the stepper motor drive, and an ALV-5000/EPP multi-τ digital photon correlator with 288 channels. Toluene was used as the index-matching fluid in the vat containing the sample cell. The samples were measured into cylindrical optical glass cells after filtration through 0.45 μm hydrophilic PVDF filters (Millipore, Billerica, MA, USA) for the removal of potent dust particles. The autocorrelation functions and hydrodynamic radii, as well as the simultaneously recorded light scattering intensity, were recorded as the average of five measurements at a goniometer angle of 90°, obtained by the cumulants method and the inverse Laplace transformation using the regularized CONTIN algorithm.

## 3. Results and Discussion

### 3.1. Synthesis and Characterization of P(LMA-co-tBMA-co-MAA) Copolymers

**Scheme 1:**
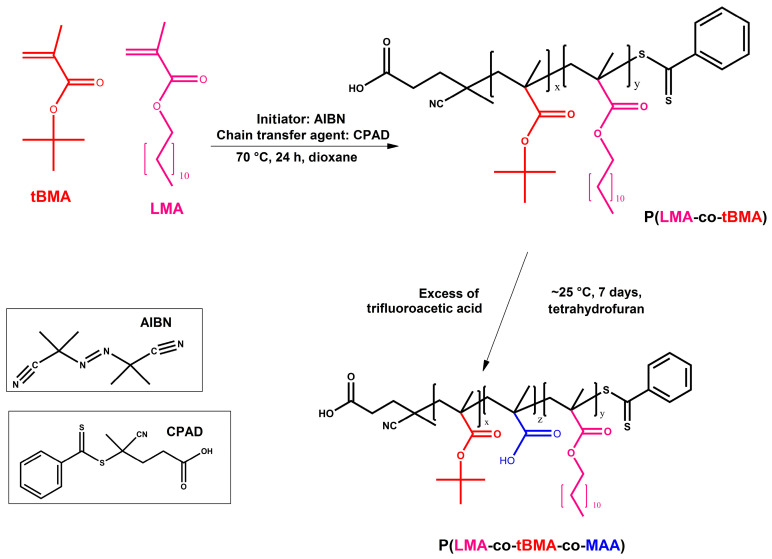
Synthesis route for P(LMA-co-tBMA-co-MAA) copolymers.

**Figure 1:**
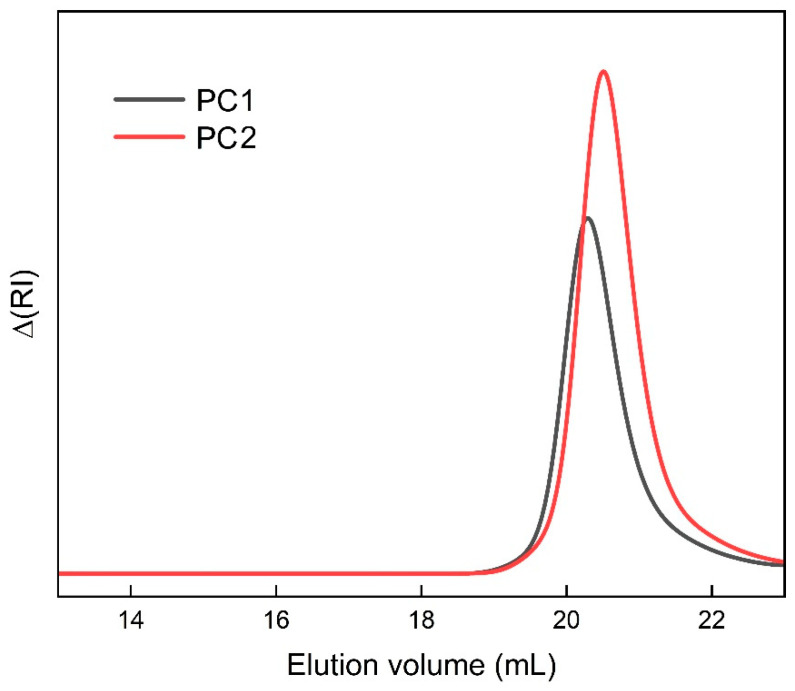
SEC traces of synthesized precursor copolymers.

Two random amphiphilic polyelectrolyte P(LMA-co-tBMA-co-MAA) copolymers were synthesized by combining the one-pot RAFT copolymerization of tBMA and LMA with follow-up selective acid hydrolysis reactions, as illustrated in Scheme 1. The polymerization conditions for the synthesis of the precursor copolymers PC1 and PC2 were chosen according to the literature [28]. CPAD is a chain transfer agent known to provide a high degree of control for the living radical polymerization of methacrylate-based monomers [29]. In addition, polymer derivatives of CPAD-mediated polymerization have shown remarkable potential for bioapplications, as CPAD confers polymer chains with carboxylic acid end groups [30,31]. The target average molecular weight numbers were deliberately selected according to the in vivo renal clearance limits for soluble polymers which have been explored for nanotherapeutic investigations [32,33]. The RAFT technique is compatible with a wide range of monomers; however, there is a known difference in polymerization rate and solubility amongst MAA, LMA and tBMA monomers that may lead to the production of less homogenous copolymers [21,34]. Therefore, a two-step process of polymerization and post-modification was chosen. The polymerization reaction yield was close to 95%. SEC was utilized to determine the apparent average molecular weights (M_w_) and the respective dispersity indexes (M_w_/M_n_) of the copolymers. The measured values are summarized in Table 1, and SEC traces of the PCs are depicted in Figure 1. The graphs show the differential refractive index variation as a function of elution volume. Single monomodal and relatively symmetrical peaks are observed, indicating the absence of unreacted monomers, while the M_w_ values are almost identical to the theoretically calculated ones (10,000 g/mol). The dispersities are within the range normally obtained from living RAFT random copolymerization, further confirming the narrow molecular weight distributions and controlled polymerization scheme [35].

**Figure 2:**
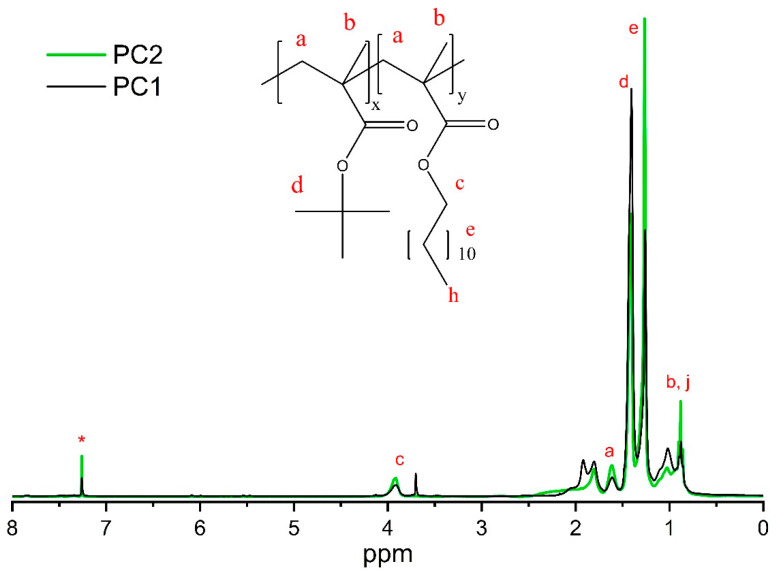
^1^H NMR spectra of PC1 and PC2 in CDCl_3_ (letters label different types of protons based on their chemical shift while solvent protons are denoted with an asterisk).

Composition distribution and chemical structure verification were attained by ^1^H NMR spectroscopy. Figure 2 shows the superimposed ^1^H NMR spectra of CP1 and CP2 while providing the peak assignments for the respective protons of the polymers’ chemical structures according to data from the literature. The monomer percent ratio, presented in Table 1, was calculated based on the estimated integration of the signals at δ 1.26 ppm (e), correlating to LMA pendant chain methylene protons [36], and δ 1.43 ppm (d), correlating to tBMA tertiary butyl group protons [37]. The obtained polymer compositions reflect the initial monomer mole ratio feed (a deviation of 2–3% was observed) for both cases of copolymerization, indicating the indeed random positioning of the two monomers along the polymer chain. By dividing the apparent average molecular weight values by the average molar mass of the repeat units, apparent polymerization degrees of 55 and 44 were calculated for PC1 and PC2, respectively. Considering the absence of alkene peaks at δ 4–6.6 ppm, this also evidenced complete monomer conversion. Together, the SEC and ^1^H NMR results confirm the well-controlled RAFT copolymerization with predictable chain length and compositions.

**Figure 3:**
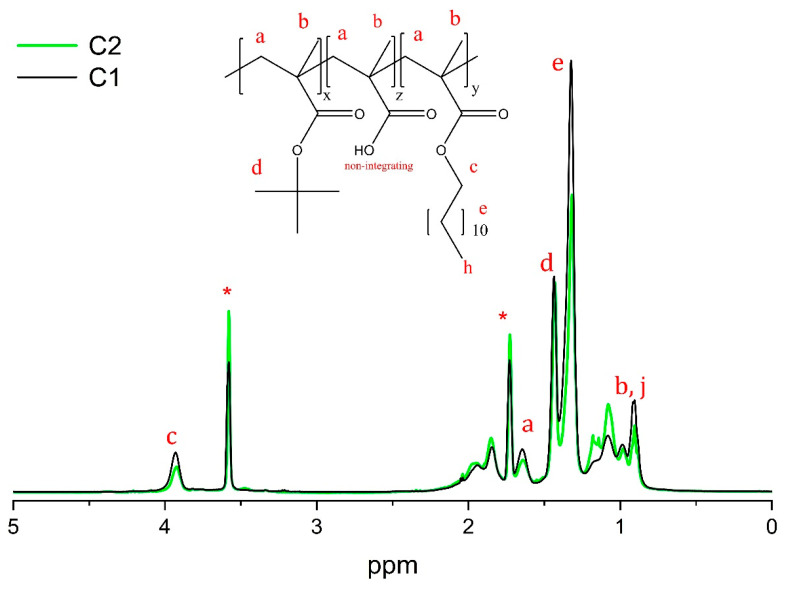
^1^H NMR spectra of C1 and C2 in THF-d8 (letters label different type of protons based on their chemical shift while solvent protons are denoted with an asterisk).

After evaluating the success of the polymerization reactions, the PCs were partially modified to their amphiphilic polyelectrolyte analogs. Regardless of the preservation of a level of tBMA segments while following facile synthetic steps, the PCs were subjected to acid hydrolysis under mild conditions. A vast collection of MAA copolymers in the literature have been prepared via the hydrolysis of precursor tBMA analogs [8,37,38,39]. The choice of reaction solvent essentially impacts the reaction efficiency, since the progressive conversion of tertiary butyl groups to carboxylic acid groups will alter the polymer chain molar mass as well as its polarity and the distribution of polar/nonpolar segments, resulting in different chain conformations that may cause steric hindrance to the ongoing hydrolysis reaction. Given the copolymers’ pronounced hydrophobic nature, with a minimum of 32% hydrophobic content (based on LMA segments) remaining invariant through the process, a low-polarity solvent which was also compatible with polar MAA was required. THF is a weakly polar aprotic solvent that also serves as a hydrogen bond acceptor, able to form hydrogen bond links with PMAA, so despite the slight polarity contrast, it tends to solubilize MAA. The solubilization of MAA homo-oligomers in THF depends on concentration, with reported good solubility at 8 mg/mL [40]. However, predicting solubility becomes challenging when immiscible comonomers are included alongside PMAA units. Even though the literature includes examples of amphiphilic PtBMA block copolymers, where the total transformation of the tertiary units to carboxylic acids units happens through hydrolysis in THF [37,41], amphiphilic PLMA-b-PMAA block copolymers have been reported to aggregate in THF solutions [42]. Thus, assuming that hydrolysis would reach a conversion limit, the reaction was carried out with reagent quantities and conditions in accordance with the complete conversion of tBMA units. Notably, the reaction solution was translucent throughout the whole process, and the obtained product yield was close to 100%. As a first sign of the presence of MAA domains in the copolymers’ structures, the copolymers showed decreased solubility in CDCl_3_; hence, the chemically modified copolymers’ compositions were determined by ^1^H NMR spectroscopy in THF-d8, as shown in Figure 3. The hydroxyl protons of the carboxylic acid groups undergo rapid exchange with nearby protons, relating to a non-integrating peak that often disappears. Therefore, calculations were correspondingly based on the integral estimation of the characteristic proton chemical shifts at δ 1.26 and 1.43 pmm, with the latter appearing remarkably weakened compared to the former precursor copolymer spectra (Figure 2), reflecting the diminished number of tertiary butyl groups in the hydrolyzed copolymers. Taking into account the immutable consistency of LMA before and after hydrolysis, estimations revealed a similar, relatively satisfactory conversion for both PC1 (71%) and PC2 (64%). The final copolymer–monomer ratio percentages are presented in Table 1.

As expected, several tBMA segments remain unaffected in both cases. Interestingly, the proportion is almost identical for the two PCs that differ in comonomer content. It is possible that the slightly lower conversion of PC2 is attributable to amplified steric hindrance due to the higher percentage of LMA long pendant chains. On the assumption that the apparent polymer chain length is built from approximately 60 to 40 units, the dodecyl LMA pendant chains shape short branches on the linear main chain structure, inducing an architecture of higher complexity. Furthermore, it has been reported that regarding tBMA homopolymers that present M_w_ between a range of 13,200, 15,600, 28,700 and 53,500 g/mol, when hydrolysis happens in dioxane with HCl, hydrolysis ratio remains constant at 77.87 ± 1.65%, an occurrence that does not seem to apply in the present occasion, considering that a wider deviation in hydrolysis ratio is observed alongside a rather low deviation in average M_w_. Nonetheless, such conclusions are hypothetic due to the various interactions between multiple polymer chains of random segment distributions [24].

**Figure 4:**
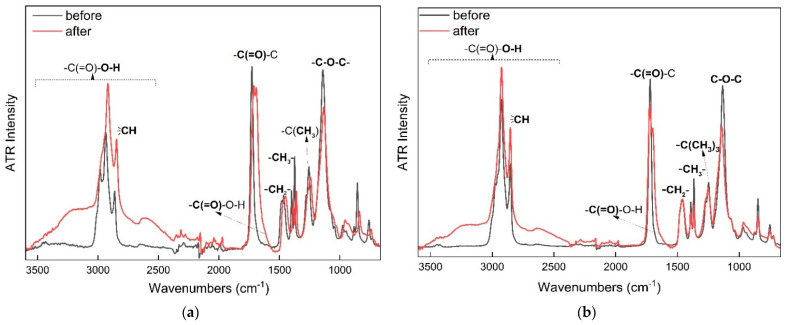
FTIR spectra of solid (**a**) copolymer 1 and (**b**) copolymer 2 before and after hydrolysis.

FTIR spectroscopy was also utilized to verify the copolymers’ transformations. Figure 4a,b shows representative spectra of PC/C 1 and 2 before and after hydrolysis. Analogous spectral variations occur for both hydrolyzed copolymers relative to the distinguishable peak positions in the absorbance spectra of the PMAA copolymers [43,44]. The most obvious of which is the wide and strong absorption peak appearing between 2500 and 3550 cm^−1^, assigned to the stretching vibrations of carboxylic hydroxyls –C(=O)**O–H.** The red shift and slight splitting of the sharp peak near 1723 cm^−1^, assigned to carbonyl –**C=O** stretch modes, is a characteristic variation as well, reflecting the partial conversion of the ester groups of the methacrylate repeating units to carboxylic acid (from –**C(=O)**–O–C to –**C(=O)**–O–H). Additionally, the intensity of the cleaved peak at 1370–1390 cm^−1^ and the peak at 1200–1255 cm^−1^, ascribed to -CH_3_ common bending vibrations and C-C skeletal vibrations of tertiary butyl groups, respectively, is decreased [45,46]. A somehow decreased intensity can also be noticed in the sharp absorption peak near 1134 cm^−1^, assigned to C-O-C stretching vibrations, further signifying the loss of ester groups. On the contrary, even though normalization was not applied to the spectra, the intensity of the peak at 1457 cm^−1^, associated with -CH_2_- bending vibrations, seems to not decrease after hydrolysis, marking the integrity of the polymer chains’ backbones [47]. The above observations indicate that solid-state analysis corroborates the results from the ^1^H NMR measurements.

### 3.2. Self-Assembly Studies of P(LMA-co-tBMA-co-MAA) Copolymers in Aqueous Solutions

#### 3.2.1. CAC Determination

**Figure 5:**
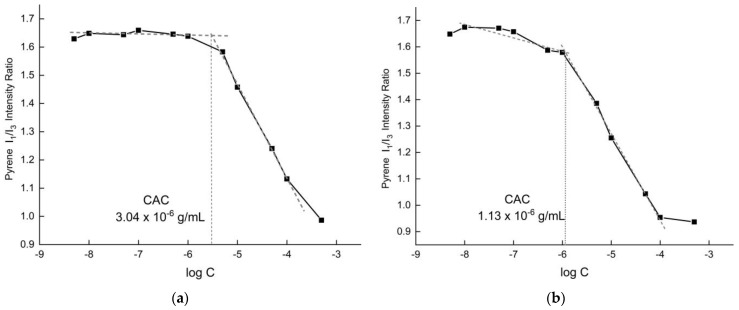
CAC determination in aqueous solution at pH = 7 for (**a**) C1 and (**b**) C2 copolymers.

Next, we were interested in the self-assembly behavior of the hydrolyzed copolymers, which were expected to exhibit amphiphilic and polyelectrolyte characteristics. The formation of self-assembled aggregates in aqueous solutions was confidently anticipated due to the significant fraction of hydrophobic segments in the copolymer chains. Direct dissolution in aqueous media was not feasible within 24 h; therefore, all self-assembly experiments were conducted via the organic solvent evaporation protocol. CAC is defined as the minimum concentration at which self-assembly is thermodynamically favored instead of molecular dispersion, forcing aggregates to appear. This characteristic value apparently exists for every self-assembled system and is crucial in terms of biological nanotherapeutic applicability, since it reflects the stability at extreme dilutions associated with the presence of the nanoassembly in the bloodstream [48]. Fluorescence spectra, based on the fluorescence intensity and wavelength fluctuations of a fluorescent probe in the solvent or aggregation phase, can sensitively detect CAC. Fluorescence probes exhibit characteristic emission or excitation spectra indicative of the polarity of their surrounding micro-environment [43]. In this case, we measured the fluorescence of serial copolymer dilutions with the selective partition of the hydrophobic fluorescent probe pyrene. It is commonly reported that the ratio of the intensities of the first and third vibration band I_1_ (λ ≈ 373 nm)/I_3_ (λ ≈ 383 nm) in the emission spectrum of pyrene increases when pyrene accumulation shifts from an apolar to a polar environment; hence, a low ratio would be a sign of the localization of the probe in the hydrophobic cores of polymer aggregates [49]. Figure 5a,b show plots of the I_1_/I_3_ ratio versus the logarithm of copolymer concentration for the C1 and C2 copolymers, respectively. CAC is determined as the concentration correlating to the intersection of the tangent dot lines on the curve. The two copolymers present equally sharp transitions, indicating that their aggregations occur in a similar manner, while, as normally observed for similar types of amphiphilic copolymers, C2, with a higher content in the hydrophobic segment, presents a lower CAC value than C1 [50]. The estimated values, which are numerically displayed within Figure 5, align with reported values from the literature involving copolymers of MAA with tBMA or LMA [22,43,51], as well as with other amphiphilic copolymers studied for bioapplications [52]. This suggests that both C1 and C2 potentially form thermodynamically stable aggregates in physiological media, possessing hydrophobic interiors in the higher concentration range, as indicated by the I_1_/I_3_ values being below 1.

#### 3.2.2. pH-Responsive Self-Assembly

**Figure 6:**
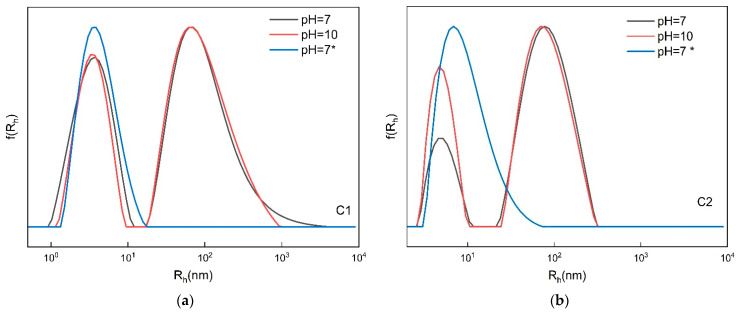
R_h_ distribution curves of (**a**) C1 and (**b**) C2 copolymers in aqueous solutions of varying pH, obtained from CONTIN analysis.

Following CAC investigation, DLS experiments were performed to further examine the physical characteristics and the apparent pH sensitivity of the aggregates formed. Measurements were first conducted in solution samples under pH 7, which were already formed by the organic solvent protocol. Then, samples of the same solution were manipulated (by the addition of HCl or NaOH aqueous solutions) to obtain solutions of pH 10, from which samples were measured or altered to obtain pH 3 solutions. At pH 3, both copolymers presented almost immediate precipitation due to the deprotonation of the -COOH groups in their MAA segments; therefore, no measurements were conducted. However, the samples were again manipulated to obtain pH 7 solutions. Clear solutions were obtained in this scenario, indicating that the precipitation was reversible by the alteration of solution pH, and were measured later. The results, including average hydrodynamic radii (R_h_), size polydispersity indexes (PDIs) and light scattered intensity (I_90_), are tabulated in Table 2, while size distribution curves from CONTIN analysis are provided in Figure 6. The measurements from the latter pH 7 solutions, obtained after the increase in pH in the precipitated solutions, are denoted with an asterisk (*). The optimal size of amphiphilic NPs varies depending on their intended application; typically structures of 5–500 nm diameter are investigated due to their repeatedly reported efficiency in drug delivery protocols [53]. NPs with diameters smaller than 5 nm face rapid clearance from the body by renal filtration and urinary excretion; however their cellular uptake has been demonstrated in vivo by multiple types of immune cells [54,55].

In general, the DLS results display nanoassemblies with a mean R_h_ in the range of 3–95 nm, while both copolymers present discrete bimodal size distributions when prepared by the organic solvent protocol. pH-dependent self-assembly is clearly observed. The lower MAA content compared to LMA and tBMA explains why the copolymer assemblies are not stable in acidic solutions even though the apparent pK_a_ of MAA is reported to be ~4.8. Over that pH value, MAA chains are supposed to present hydrophilicity due to deprotonation, and usually swelling occurs for micelles that comprise MAA coronas. Size variation, though, does not necessarily follow pH variation in an analogous manner [56,57]. This does not seem to apply in the present case either, since when the solution pH is increased from 7 to 10, an almost identical aggregate distribution occurs for both C1 and C2. However, the measured I_90_ also slightly decreases, meaning that the aggregates appear to have lower masses at higher pHs. This observation is a sign that swelling exists to a certain extent, causing the loosening of the aggregates, despite this not being translated to the aggregate size. This assumption implies that, even at pH 10, where the complete deprotonation of the -COOH groups in the MAA segments is expected, the hydrophobic interactions are still very strong; hence, the copolymers do not tend to disperse. As mentioned above, adjusting the pH value to 3 caused the collapse of the systems, as it seemed that due to the protonated MAA units, the ionization level was not enough to overcome the hydrophobic interactions, and electrostatic repulsions became inefficient in maintaining the self-assembled structures that were initially formed. Another point that needs to be discussed here concerns the detailed internal structure of the aggregates produced by the amphiphilic copolymers having random distributions of hydrophilic and hydrophobic segments. Due to this, several hydrophobic nanodomains may be formed within each aggregate, bridged by looped chain sections made up of MAA segments. Although the present data cannot verify the proposed aggregate structure, which is based solely on chemical intuition, one can expect that such structures may have different behaviors in response to changes in solution pH compared to core–shell micelles formed by amphiphilic block copolymer chains.

Returning each collapsed copolymer system to neutral/alkaline conditions induces the formation of a monomodal population of a similar size (slightly higher in the case of C2) to the minority population that existed before the pH decrease, which comprises assemblies with an average R_h_ of 3–5 nm. In parallel, PDI appreciably decreases, indicating the higher size homogeneity of the obtained aggregates. In the case of C1, this phenomenon is observed along with a decrease in I_90_. This may be attributed to the almost complete rearrangement of the aggregate towards the smaller size population range. Regarding C2, while the mean R_h_ is slightly higher than the R_h_ of the smaller population, the I_90_ increases. Similarly, this indicates that the aggregate rearranges into more compact structures of smaller sizes.

At this point, it is worth considering two factors: the copolymers’ topologies and the dissolution protocol. The copolymers comprise random distributions of hydrophilic and hydrophobic segments. Self-assembled block copolymers with hydrophilic and hydrophobic alkyl pendant blocks generally form relatively large aggregates through multichain association. In contrast, their random counterparts produce single-chain (unimer) or multichain micelles via intramolecular self-folding or intermolecular self-assembly [58]. Judging by the deviation between the R_h_ of the two populations before precipitation, it is reasonable to assume that the population of smaller sizes (contributing less to solution scattering) corresponds to unimers/single-copolymer chains. Thus, rearrangement after fixation to pH 7* induces self-folding for the C1 copolymer and self-assembly into compact multimolecular aggregates that probably comprise few molecules for the C2 copolymer. The difference between the two copolymers can be attributed to the difference in their hydrophilic–hydrophobic balance. Their diverse hydrophilic–lipophilic balances are also reflected in the measured I_90_ which is, in general, approximately five times higher for C2, where hydrophobic interactions cause densely packed aggregates. Nonetheless, the copolymers seem to follow a similar pattern in terms of pH sensitivity, taking into account the preparation protocol. The differences observed in the pH 7 and pH 7* cases evidence that the initial dissolution in THF (a much better and less selective solvent) significantly affects self-assembly behavior. It is possible that, due to the slight immiscibility of THF with MAA units, the copolymers already present a self-assembled structure in this solvent before mixing with the aqueous medium, which, after the evaporation of the organic solvent, facilitates the formation of aggregates close to the equilibrium with the participation of several copolymer chains.

#### 3.2.3. Time-Dependent Self-Assembly

**Figure 7:**
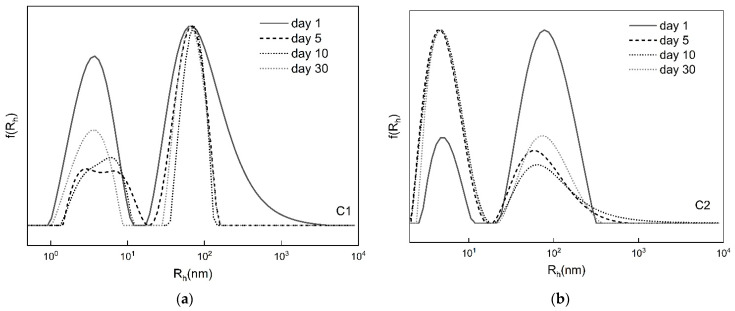
R_h_ distribution curves of (**a**) C1 and (**b**) C2 copolymers from repetitive measurements of pH 7 aqueous solutions, obtained from CONTIN analysis.

Supplementary DLS experiments were performed for the copolymer solutions at pH 7 in order to evaluate the nanosystems in terms of their stability over time. Measurements of the same sample were conducted five, ten and thirty days after the first measurement. The results are displayed in Table 3, and Figure 7 shows the size distribution curves obtained from the CONTIN analysis. The two copolymers follow an opposite self-assembly behavior pattern. For the C1 copolymer, it can be observed that its bimodal size distribution is maintained at almost the same total scattered intensity, while the multimolecular aggregates rearrange or aggregate further to form compact aggregates of a smaller size. Another possible scenario is that some of the larger aggregates that contributed to the tail of the wider peak at higher sizes (Figure 7a, day 1 curve), separate and form unimers or assemblies of fewer chains, hence the slight increase in mean R_h_. It is important to note that, since the polymers are derived from random copolymerization, not all the unimers are necessarily of equal size and composition, resulting in differences in amphiphilicity. Concerning the C2 copolymer, it can be observed that, in the span of five days, self-assembly transits the polymer into a system where more unimer structures are preferred. The bimodal size distribution is reversed in terms of relative intensities, and total I_90_, along with PDI values, decreases. The population of multimolecular aggregates (which decreases in relative intensity) presents a relatively constant R_h_. A common observation for both copolymers is that the severe variations happened between the first and fifth day, and the systems seem to present a relative equilibrium over the course of a month. A combination of chain dynamics and hydrophobic interactions seems to occur, where the more hydrophobic C2 copolymer undergoes more rearrangements to reach an equilibrium. It should be noted that there was no precipitation observed for either of the two copolymer solutions. Therefore, these copolymer systems seem to be very dynamic, and this should be correlated to their random chain architectures.

## 4. Conclusions

Random amphiphilic polyelectrolyte copolymers P(LMA-co-tBMA-co-MAA), bearing high contents of hydrophobic segments, were successfully synthesized via RAFT polymerization and post-polymerization acid hydrolysis in THF. The molecular characterization of the precursors and successor copolymers confirmed the production of relatively tailored products. Fluorescence studies revealed the copolymers’ ability to form aggregates in aqueous solutions, with a significantly low critical aggregation concentration and a hydrophobic interior, indicating their high thermodynamic stability. In addition, dynamic light scattering experiments evidenced that their self-assembly behavior was dependent on copolymer composition, pH or dissolution variations inducing the formation of unimers or mixed unimer and multimolecular nanoassemblies under different conditions. Moreover, self-assembled structures of average R_h_ ≤ 100 are observed at physiological pH, meaning that small-size structures with hydrophobic cavities and hydrophilic, negatively charged surfaces are formed. These properties could be beneficial for a series of applications, including the encapsulation and delivery of hydrophobic drugs, complexation with cationic bioactive compounds, protein/enzyme immobilization or dyes for bioimaging, alone or in combinations (e.g., for co-delivery or delivery and imaging). Future investigations could explore the affinity and coherency of the systems to specific applications and targeted therapies. For example, controlled release based on pH sensitivity could be compared to potential targeted therapy by exploiting surface functionalization, whereas high hydrodynamic resistance to precipitation in certain pH values could be evaluated for bioimaging applications. Overall, the prepared copolymers offer potential for the development of multifunctional carrier systems for nanotherapeutic applications.

## Figures and Tables

**Table 1:** Molecular characteristics of synthesized copolymers.

PC/C	M_w_ ^a^ (g/mol) (×10^4^)	M_w_/M_n_ ^a^	%wt LMA	%wt tBMA ^b^ Before Hydrolysis	%wt tBMA ^b^ After Hydrolysis
1	1.14	1.16	32	68	20
2	1.02	1.19	47	53	19

^a^ Determined by SEC before hydrolysis. ^b^ Determined by ^1^H NMR in CDCl_3_ before and in THF-d8 after hydrolysis.

**Table 2:** DLS analysis results for copolymers in aqueous solutions of different pH.

Copolymer	pH	I_90°_ (a.u.)	R_h_ (nm)	PDI
C1	7	35	95 (62%)/3 (38%)	0.531
	10	30	90 (66%)/4 (34%)	0.552
	7*	20	4	0.490
C2	7	165	82 (80%)/5 (20%)	0.542
	10	108	80 (70%)/5 (30%)	0.549
	7*	138	10	0.342

**Table 3:** DLS analysis results for repetitive measurements of pH 7 solutions over a month.

Copolymer	Day	I_90°_ (a.u.)	R_h_ (nm)	PDI
C1	1	35	95 (62%)/3 (38%)	0.531
	5	52	64 (62%)/5 (38%)	0.550
	10	46	71 (60%)/5 (40%)	0.521
	30	43	66 (60%)/3 (40%)	0.544
C2	1	165	82 (80%)/5 (20%)	0.542
	5	70	75 (36%)/5 (64%)	0.492
	10	50	79 (32%)/5 (68%)	0.476
	30	65	80 (39%)/5 (61%)	0.494

## Data Availability

All data are included in the manuscript.

## Abbreviations

The following abbreviations are used in this manuscript:

C1	Copolymer 1
C2	Copolymer 2
PC	Precursor copolymer
MAA	Methacrylic acid
LMA	Lauryl methacrylate
tBMA	Tert-Butyl methacrylate
RAFT	Reversible-addition fragmentation chain transfer
